# Malignant Transformation of a Giant Solitary Trichoepithelioma With Extensive Craniofacial Invasion: An Unusual Case Report and Review of Literature

**DOI:** 10.7759/cureus.112458

**Published:** 2026-07-11

**Authors:** Salapu Ramesh Babu, V Kishore Reddy Bojja, P Vinodhini, Manilal B, Narendra H

**Affiliations:** 1 Surgical Oncology, Sri Venkateswara Institute of Medical Sciences, Tirupati, IND

**Keywords:** anterolateral thigh (alt) flap, craniofacial resection, desmoplastic trichoepithelioma, malignant trichoepithelioma, skin adnexal neoplasms

## Abstract

Trichoepithelioma is a rare benign follicular adnexal neoplasm with hair follicle differentiation, most commonly occurring on the face. Malignant transformation is exceptionally uncommon, with fewer than 10 cases reported in the literature, all arising in patients with multiple familial trichoepitheliomas. To the best of our knowledge, this is the first reported case of malignant transformation of a giant solitary trichoepithelioma with extensive craniofacial invasion. We report the case of a 52-year-old man who presented with a rapidly progressive ulcerative lesion over the right side of the face, associated with restricted ocular movements and diminished vision. He had undergone excision of a similar lesion at the same site two years earlier, which was histopathologically diagnosed as trichoepithelioma. Radiological imaging demonstrated a locally aggressive soft-tissue mass with extension into the orbit, adjacent musculature, and underlying facial bones. Histopathological examination revealed infiltrative nests and cords of basaloid tumour cells within a desmoplastic stroma, with perineural, muscle, and bone invasion, consistent with malignant transformation of trichoepithelioma. The patient underwent radical craniofacial resection with histologically negative margins followed by reconstruction. He remains disease-free at 30 months, with serial clinical examinations every three months and contrast-enhanced computed tomography every six months. This case highlights the importance of considering malignant transformation in recurrent or rapidly enlarging trichoepitheliomas and signifies the role of complete surgical excision and long-term surveillance.

## Introduction

Trichoepithelioma is a rare benign follicular adnexal neoplasm with hair follicle differentiation that most commonly arises on the face and scalp. Clinically, it presents as a small, painless, slow-growing papule or nodule and has been traditionally described as solitary, multiple familial, desmoplastic, and giant solitary variants. [1] Although trichoepithelioma generally follows an indolent clinical course with an excellent prognosis, malignant transformation is exceedingly rare [2,3].

The term malignant trichoepithelioma has historically been used in a few case reports to describe malignant transformation arising within a pre-existing trichoepithelioma, particularly in patients with multiple familial trichoepitheliomas. However, it is not recognized as a distinct entity in the current WHO Classification of Skin Tumours, and the diagnosis relies on clinicopathological correlation [3-7].

Malignant transformation of trichoepithelioma is exceptionally rare, with fewer than 10 cases reported in the literature, all occurring in patients with multiple familial trichoepitheliomas [8]. To the best of our knowledge, malignant transformation arising in a solitary, non-familial trichoepithelioma has not previously been reported.

Given its rarity and considerable clinicopathological overlap with other basaloid adnexal neoplasms, malignant transformation of solitary trichoepithelioma poses a significant diagnostic challenge. We report what appears to be the first case of malignant transformation of a giant solitary trichoepithelioma with extensive orbital and craniofacial invasion, successfully managed by radical craniofacial resection with histologically negative margins and reconstruction, with no clinical or radiological evidence of recurrence after 30 months of follow-up.

## Case presentation

A gentleman aged 52 years with good performance status (Eastern Cooperative Oncology Group (ECOG) I) presented to the surgical oncology outpatient department with an ulcer over the right side of the face for the past three months. The lesion had initially been small but showed rapid progression in size. He also reported significant restriction in ocular movements and diminished vision in the right eye. Notably, the patient had undergone surgical excision of a superficial lesion at the same site two years earlier at an outside institution. A printed histopathology report was available at presentation, and the original histopathology slides and paraffin blocks were subsequently retrieved and reviewed for diagnostic correlation. Histopathological review of the slides and paraffin blocks confirmed trichoepithelioma.

On clinical examination (as shown in Figure 1), a 7×5 cm ulcerative lesion with rolled-out margins and a slough-covered base was observed over the right temporal region, associated with purulent discharge and appeared to involve underlying structures, with clinical suspicion of bony erosion. Ocular examination revealed a marked restriction of right eye movements. There was no palpable lymphadenopathy in the cervical, auricular, or parotid regions.

**Figure 1:**
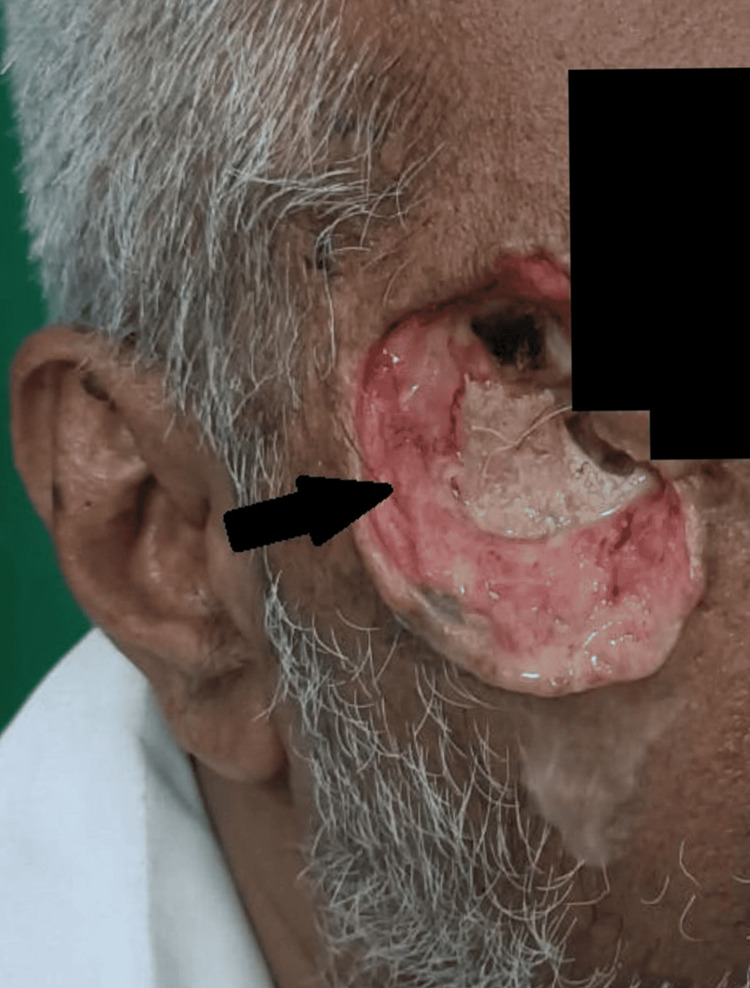
Pre-operative clinical image of the patient (black arrow showing the ulcerative lesion with visible bone at the base on the right side of the face)

Histopathological examination of the preoperative incisional biopsy revealed irregular nests and cords of basaloid tumour cells within a desmoplastic stroma, exhibiting hair germ differentiation, focal peripheral palisading, and occasional papillary mesenchymal bodies. The tumour lobules were surrounded by a fibrous stroma, and infiltrative growth extending to the biopsy margins and base was identified. These findings, together with a review of the previous trichoepithelioma, support malignant transformation of a solitary trichoepithelioma.

A radiological assessment was performed to evaluate the extent of the disease. Contrast-enhanced computed tomography (CECT) reveals a soft-tissue-density lesion that is medially extending into extraconal fat, infiltrating the lateral rectus and lacrimal gland, and focally infiltrating the right temporalis muscle at the superior end. It extends inferiorly up to the cheek and laterally into the zygomatic attachment of the zygomaticus major and minor muscles. The right maxillary sinus is directly communicating with the lesion base. Erosion of the right zygomatic body and zygomatic arch, the anterior and lateral walls of the right maxilla, and the floor of the right orbit was noted, indicating extensive local bony invasion.

Contrast-enhanced magnetic resonance imaging (as shown in Figure 2a-d) demonstrated an enhancing lesion involving the right cheek extending into the inferolateral orbit with invasion of the temporalis muscle, zygomatic bone, maxillary sinus, orbital floor and lateral rectus muscle without intracranial extension.

**Figure 2:**
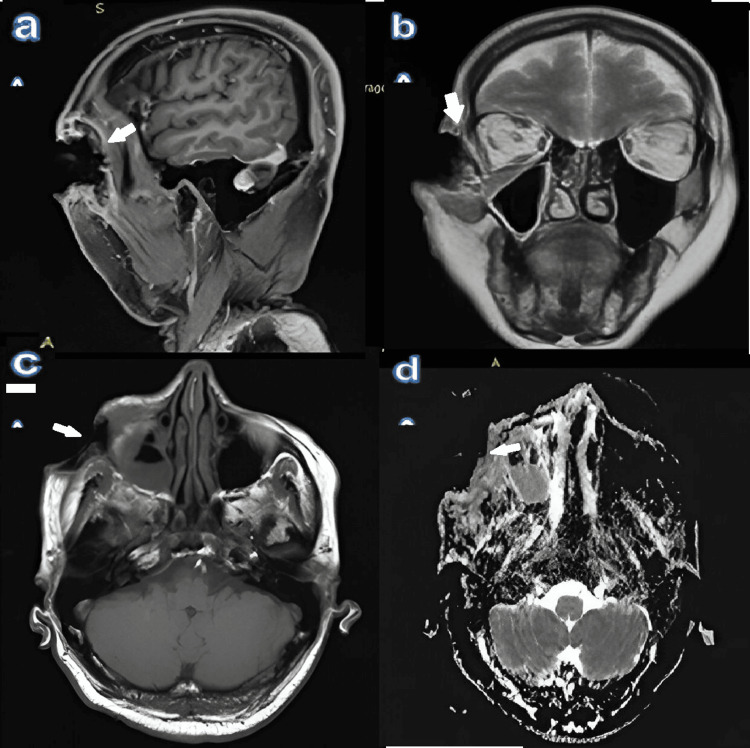
Contrast-enhanced MRI of the patient showing an altered signal intensity lesion (white arrow) that is heterogeneously hyperintense on T2: (a) sagittal plane; (b) coronal plane; (c) axial plane; and (d) diffusion-weighted imaging.

PET-CT shows a metabolically active soft tissue density lesion in the right periorbital region involving pre-antral and zygomatic space with erosion of the anterior wall of the maxillary sinus and zygomatic bone with extension into the sinus. Superiorly extending and involving the lateral aspect of the lower eyelid, the lateral canthus extends into the orbit. No evidence of metabolic activity elsewhere in the body.

Following multidisciplinary discussion, the patient underwent radical craniofacial resection. Intraoperatively (as shown in Figure 3a-c), a 7x5 cm ulcerative lesion was noted, involving the right temporal region and the lateral aspect of the right eye that was infiltrating the inferior and lateral orbital rims. The temporalis muscle is dissected from the periosteum. A key burr hole was placed over the temporal bone corresponding to the middle cranial fossa. To do a frontotemporo-orbitozygomatic craniotomy, the frontal and zygomatic bones were undercut, and the sphenoid ridge was nibbled. The meningo-orbital band was coagulated and cut. Bone incisions were made across the maxilla, frontal nasal process, zygoma, and inferior orbital rim, and the palatal mucosa were elevated. The optic nerve was ligated and cut, and the contents of the orbit were reflected. The lesion was removed locally with a 2 cm margin, and hemostasis was secured. There was a minimal cerebrospinal fluid (CSF) leak near the cut end of the optic nerve. Local fat and fascia were used to seal the CSF leak. The anterolateral thigh flap harvested from the right thigh was used for reconstruction, and the flap was closed over the corrugated rubber drain (CRD) drain. The donor site is covered with a split skin graft from the left thigh.

**Figure 3:**
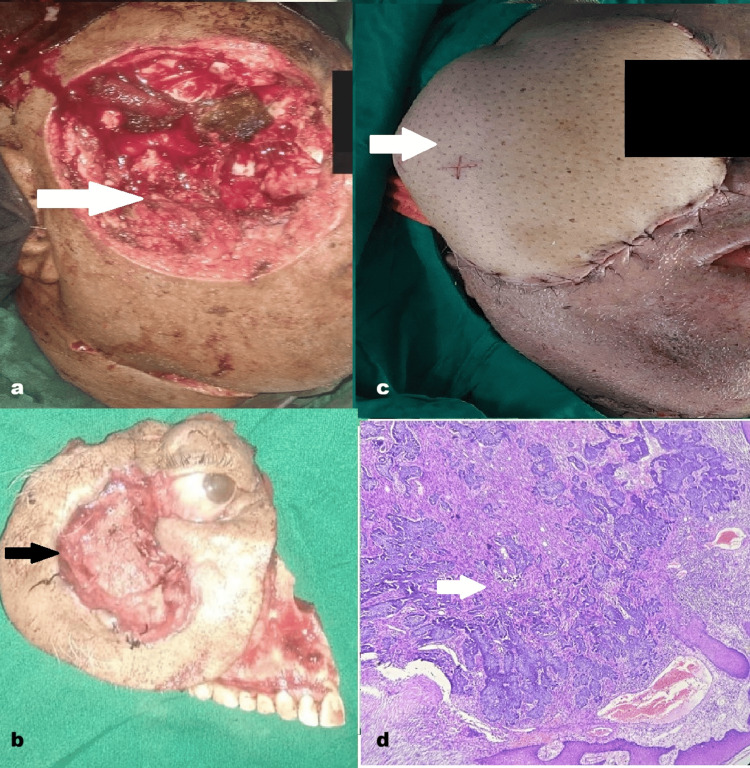
Images of the patient: (a) Intraoperative image of the patient after resecting the tumour (white arrow showing the postoperative tumour bed); (b) resection specimen (black showing the lesion with 2 cm margin); (c) postoperative image of the patient after reconstruction (white arrow showing the ALT flap); (d) post HPE microphotograph - hematoxylin and eosin stain (10x). (white arrow showing basaloid tumour cells in nests with granular chromatin, nucleoli, and amphophilic cytoplasm). ALT: Anterolateral thigh; HPE: histopathological examination.

Histopathological examination (as shown in Figure 3d) of the resected specimen demonstrated an ulcerated lesion composed of infiltrative nests, sheets, and cords of basaloid tumour cells within a desmoplastic stroma. The tumour cells exhibited hyperchromatic nuclei with granular chromatin, conspicuous nucleoli, and a moderate amount of amphophilic cytoplasm. The foci of tumour necrosis and myxoid stromal change were identified. Perineural invasion (small unnamed nerve) was present, with extension of the tumour into adjacent skeletal muscle bundles and bone trabeculae, confirming locally invasive behaviour. All resection margins were free of tumour on microscopic examination. Based on the characteristic histomorphological features and clinicopathological correlation, a diagnosis of malignant transformation of a giant solitary trichoepithelioma was established.

The postoperative course was uneventful, and the patient recovered well with a viable flap. The drain was removed on postoperative day six, and the patient was discharged in a stable state with a healthy flap. The case was reviewed at a multidisciplinary tumour board. In view of complete surgical excision with histologically negative margins (>1 cm) and the absence of established guidelines supporting adjuvant radiotherapy for completely resected lesion, a consensus decision was made to proceed with close clinical and radiological surveillance without adjuvant treatment. He is on a personalized surveillance protocol consisting of a clinical examination every three months and CECT every six months. At 30 months of follow-up, there is no evidence of clinical or radiological recurrence (as shown in Figure 4).

**Figure 4:**
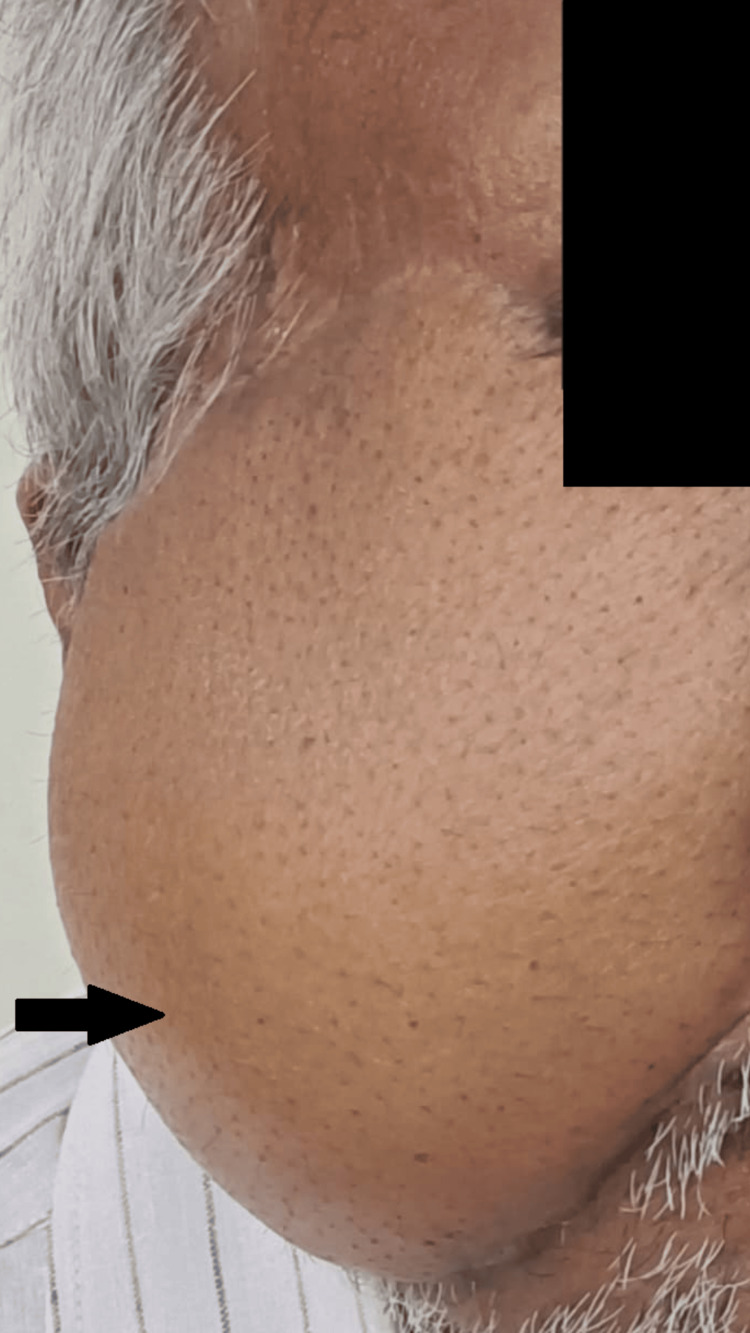
Clinical image of the patient (black arrow showing the postoperative site with a healed ALT flap and no signs of recurrence) at 30 months follow-up.

## Discussion

Trichoepithelioma was first described by Brooke in 1892 as epithelioma adenoides cysticum and is now recognized as a benign follicular adnexal neoplasm with hair follicle differentiation. Subsequent studies by Montgomery, Lever, and Pinkus further established its follicular origin and histogenesis. Historically, trichoepithelioma has been described in four clinicopathological variants: solitary, multiple familial, desmoplastic, and giant solitary trichoepithelioma. Multiple familial trichoepithelioma is associated with autosomal dominant germline CYLD mutations (chromosome 16q12-q13), resulting in dysregulated NF-κB signalling and occurring as part of Brooke-Fordyce syndrome [1,8-13].

In contrast, solitary trichoepithelioma, first described by Zeligman in 1960, is a sporadic lesion without a well-defined molecular pathogenesis. Most lesions measure <1 cm, whereas lesions ≥2 cm are historically referred to as giant solitary trichoepithelioma, a rare variant first reported by Czernobilsky in 1972 [14-16].

The term "malignant trichoepithelioma" has historically been used in published case reports to describe carcinoma arising within a pre-existing trichoepithelioma [3,4]. However, the current WHO Classification of Skin Tumours does not recognize malignant trichoepithelioma as a distinct diagnostic entity or define its formal diagnostic criteria. Instead, malignant follicular neoplasms are classified under entities such as trichoblastic carcinoma, trichilemmal carcinoma, pilomatrix carcinoma, and malignant proliferating trichilemmal tumour [5,6]. Consequently, the diagnosis relies on comprehensive clinicopathological correlation rather than WHO-defined criteria. The mechanism underlying the malignant transformation of trichoepithelioma remains poorly understood. Owing to the extreme rarity of this entity, no specific molecular alterations or genetic mutations have been consistently identified, particularly in solitary trichoepithelioma. Although malignant transformation has been described in recurrent or long-standing lesions, the underlying pathogenesis remains unknown [8].

The principal diagnostic challenge is distinguishing malignant transformation of a solitary trichoepithelioma from other basaloid adnexal neoplasms, particularly basal cell carcinoma and trichoblastic carcinoma. Trichoepithelioma typically demonstrates follicular differentiation, horn cysts, papillary mesenchymal bodies, and minimal cytological atypia, whereas malignant transformation is characterized by infiltrative growth, stromal desmoplasia, cytological atypia, and invasion of adjacent soft tissue, skeletal muscle, bone, or perineural structures [5-7,17].

Dermoscopy has emerged as a useful non-invasive adjunct in the evaluation of trichoepithelioma and may facilitate differentiation from basal cell carcinoma by demonstrating characteristic features such as multiple milia-like cysts, fine arborizing vessels, and white rosettes, although overlap exists. Recent reports have highlighted its value in improving preoperative diagnostic accuracy for follicular adnexal tumours [18,19].

In the present case, histopathological review of the slides and paraffin blocks from the previous excision confirmed trichoepithelioma. Correlation with the recurrent biopsy and resection specimens, which demonstrated infiltrative basaloid proliferation with follicular differentiation, perineural invasion, skeletal muscle invasion, and bony infiltration, supported the diagnosis of malignant transformation of a solitary trichoepithelioma. Although immunohistochemistry can be useful in distinguishing malignant transformation of a solitary trichoepithelioma from basal cell carcinoma and trichoblastic carcinoma, it was not performed in the present case because the diagnosis was established based on characteristic histo-morphological findings and clinicopathological correlation, including a review of the previous trichoepithelioma. Furthermore, there are currently no established guidelines recommending routine immunohistochemistry for the diagnosis of malignant transformation of a solitary trichoepithelioma.

Hunt and Abell first described malignant transformation in hereditary trichoepithelioma, followed by Lee et al., both reporting malignant transformation in patients with multiple familial trichoepitheliomas [3,4]. In contrast, our patient developed malignant transformation in a recurrent solitary, non-familial trichoepithelioma with extensive orbital, muscular, and bony invasion. This case therefore broadens the clinicopathological spectrum of trichoepithelioma and highlights the importance of considering malignant transformation in recurrent or rapidly enlarging solitary trichoepitheliomas.

Both CECT and CE-MRI accurately delineated the extent of local disease, demonstrating invasion of the orbit, temporalis muscle, zygomatic bone, orbital floor, and maxillary sinus without intracranial extension. Whole-body PET-CT excluded regional nodal and distant metastatic disease. Although metastatic potential has been reported, there is currently no established consensus regarding routine sentinel lymph node biopsy in clinically node-negative patients because of the limited number of reported cases. In our patient, sentinel lymph node biopsy was not performed because there was no clinical or radiological evidence of nodal involvement.

Complete surgical excision with histologically negative margins remains the cornerstone of treatment. Radical craniofacial resection achieved histologically negative margins exceeding 1 cm. Following multidisciplinary tumour board review, adjuvant radiotherapy was not recommended because complete surgical excision had been achieved and there is no established evidence supporting routine postoperative radiotherapy after margin-negative resection. The patient was placed on a personalized surveillance protocol comprising clinical examination every three months and CECT every six months. At 30 months of follow-up, there is no evidence of clinical or radiological recurrence.

Limitations

Dermoscopy was not performed in the present case because the lesion was clinically advanced with ulceration and extensive local invasion, necessitating tissue diagnosis by biopsy and radiological evaluation. Immunohistochemistry was not performed because the diagnosis was established based on characteristic histomorphological findings, review of the previous trichoepithelioma, and clinicopathological correlation. Sentinel lymph node biopsy was not performed because there was no clinical or radiological evidence of regional nodal involvement, and there are currently no established recommendations supporting its routine use in this rare entity. Radiotherapy was not given because complete surgical excision with histologically negative margins had been achieved, there is no established consensus supporting its routine use in margin-negative disease, and the potential risk of neurovascular toxicity outweighed the anticipated benefit.

## Conclusions

This case illustrates an exceptionally rare malignant transformation arising in a giant recurrent solitary trichoepithelioma with extensive craniofacial invasion. Although immunohistochemistry was unavailable, the diagnosis was supported by review of the previous trichoepithelioma, characteristic histomorphological features, and clinicopathological correlation. To the best of our knowledge, previously reported cases of malignant transformation have occurred in patients with multiple familial trichoepitheliomas; this appears to be the first reported case arising from a giant solitary lesion. Recurrent or rapidly enlarging trichoepitheliomatous lesions should prompt a careful histopathological reassessment to exclude important differential diagnoses, including basal cell carcinoma and trichoblastic carcinoma. Complete surgical excision with histologically negative margins remains the cornerstone of treatment, while long-term clinical and radiological surveillance is essential because of the risk of local recurrence and the potential for metastasis.
